# Acquired Amegakaryocytic Thrombocytopenia Associated With Autoimmune Hemolytic Anemia

**DOI:** 10.7759/cureus.27315

**Published:** 2022-07-26

**Authors:** Naoto Ikeda, Yuki Hisano, Takayuki Kamao, Masatoshi Uno, Takaaki Mizushima

**Affiliations:** 1 Internal Medicine, Kaneda Hospital, Okayama, JPN

**Keywords:** autoimmune disease, thrombopoietin, autoimmune hemolytic anemia, amegakaryocytic thrombocytopenia, thrombocytepenia

## Abstract

Acquired amegakaryocytic thrombocytopenia (AATP) is a thrombocytopenic disorder characterized by a decrease in megakaryocytes in the bone marrow. AATP is effectively treated with immunosuppressive therapy. We report a case of a 68-years-old male referred to us due to purpuric lesions on the extremities and was noted to be thrombocytopenic. Bone marrow biopsy showed AATP with autoimmune hemolytic anemia (AIHA). Only two cases of AATP associated with AIHA have been reported. AATP should be differentiated carefully from other causes of peripheral destruction of platelets, such as immune thrombocytopenia (ITP).

## Introduction

Acquired Amegakaryocytic thrombocytopenia (AATP) is a blood disorder characterized by a severe form of thrombocytopenia. AATP has marked thrombocytopenia with a selective absence of megakaryocytes and normal myeloid/erythroid precursors [1]. The exact prevalence of AATP is unknown. The incidence rate is estimated to be higher than reported because many cases are misdiagnosed as immune thrombocytopenia (ITP) [2]. Immunosuppressive treatment of AATP has been reported in case reports with steroids, cyclosporine, immunoglobulins, antithymocyte globulin, and rituximab [3-7]. Thrombopoietin (TPO) receptor agonists are also effective, and hematopoietic stem cell transplantation is performed in refractory cases [8,9]. We present a rare case of AATP associated with autoimmune hemolytic anemia (AIHA).

## Case presentation

**Figure 1:**
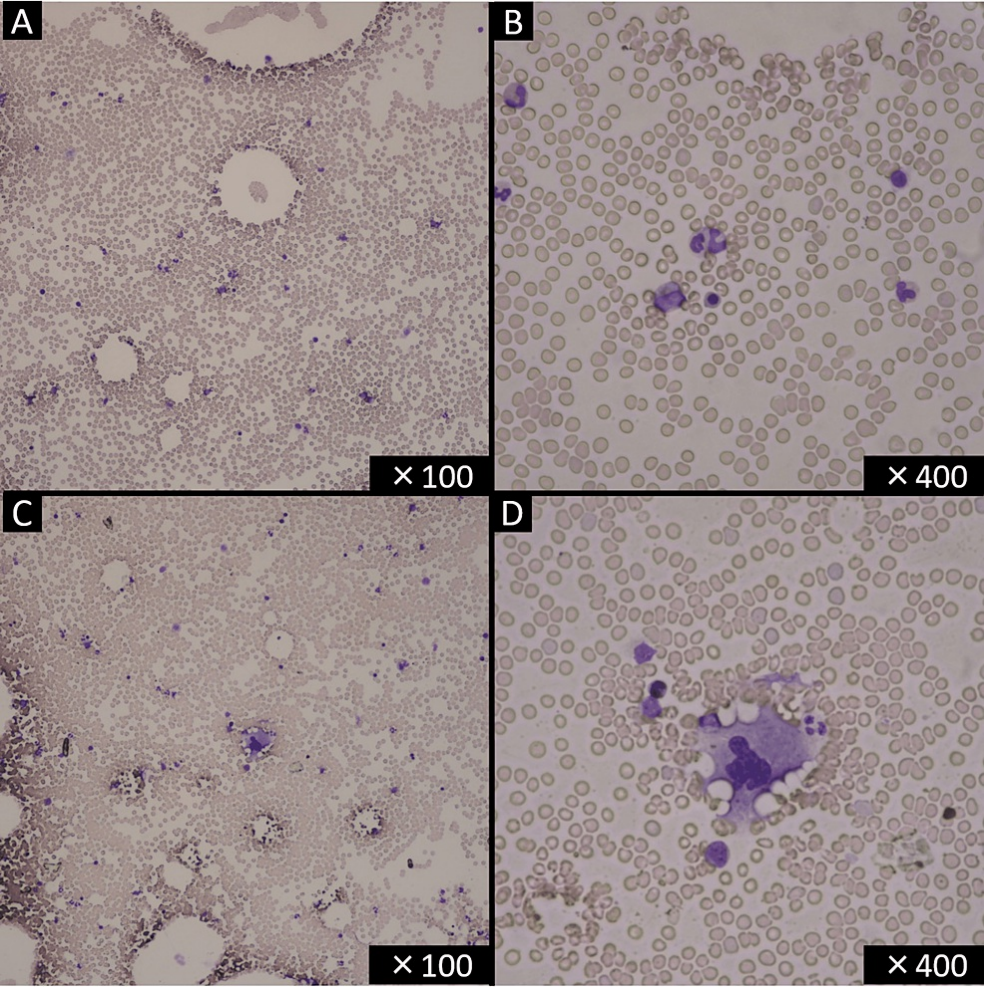
Bone marrow smear on the day of admission showed no megakaryocytes (A, B). A megakaryocyte appeared on the seventh day of admission (C, D) (May-Giemsa staining).

A 68-year-old Asian male with a past medical history of stroke, diabetes mellitus, and Helicobacter pylori gastritis visited his family doctor complaining of purpura on his limbs. He took clopidogrel, voglibose, repaglinide, metformin, sitagliptin, dimethylpolysiloxane, pantothenic acid, and sucralfate. His family doctor advised him to stop antiplatelet medication (clopidogrel). However, the purpura on his arm and legs did not resolve, and he revisited his family doctor. His hematology examination showed a platelet count of 3,000/μL, and he was referred to our hospital. Initial laboratory examination was notable for hemoglobin of 9.5 g/dL, platelet count of 2,000/µL, and an elevated reticulocyte count of 4.21% (Table 1). Vital signs were notable for tachycardia with a heart rate of 101 beats per minute, blood pressure of 131/75 mmHg, oxygen saturation of 99% on room air, and body temperature of 36.4°C. Examination of his bone marrow smear revealed no megakaryocytes with no atypical cells or blast proliferation (Figure 1A, 1B, 2A). No schistocyte was found in the peripheral blood smear. Haptoglobin was decreased, indicating hemolytic findings, and direct/indirect Coombs tests were negative. Rheumatology workup revealed rheumatoid factor positive, cyclic citrullinated peptide (CCP) antibody negative, anti-nuclear antibody (ANA) negative, dsDNA antibody negative, Sjogren's syndrome type A (SS-A) antibody negative, Sjogren's syndrome type B (SS-B) antibody negative, myeloperoxidase anti-neutrophil cytoplasmic antibody (MPO-ANCA) negative, and Proteinase 3 anti-neutrophil cytoplasmic antibody (PR3-ANCA) negative (Table 2). The patient was started on 1mg/kg of prednisolone for suspected AATP. He was treated twice with platelet transfusion, resulting in a mild increase in his platelet count. Another test bone marrow smear on the seventh day of admission showed a few megakaryocytes (Figure 1C, 1D, 2B). Clinical symptoms of purpura got better, and treatment with prednisolone was continued. The prednisolone dose was reduced to 0.5 mg/kg on the 15th day of admission because he had a history of diabetes mellitus. On the 16th day of admission, the laboratory test showed a platelet count of 39,000/μL (Figure 3).

**Table 1:** Laboratory examination and bone marrow examination on admission. MCV: mean corpuscular volume, MCHC: mean corpuscular hemoglobin concentration

Laboratory examination	On admission		Reference
White blood cells	7.8	×10³/μL	3.3-8.6
Red blood cells	3.05	×10⁶/μL	4.35-5.55
Hemoglobin	9.5	g/dL	13.7-16.8
Hematocrit	27.9	%	40.7-50.1
MCV	91.5	fl	83.6-98.2
MCHC	34.1	%	31.7-35.3
Platelet count	2	×10³/μL	158－348
Neutrophil	73.6	%	45-75
Lymphocyte	15.6	%	20-45
Monocyte	8.5	%	1.0-6.0
Eosinophil	1.8	%	1.0-5.0
Basophil	0.5	%	0-1.0
Bone marrow examination			
Number of nucleated cells	5.1	×10⁴/μL	10-20
Megakaryocyte	0	%	0.1-1.0
Myeloid/Erythroid (M/E) ratio	2.09		1.5-3.3
Dysplasia	(-)		(-)

**Figure 2:**
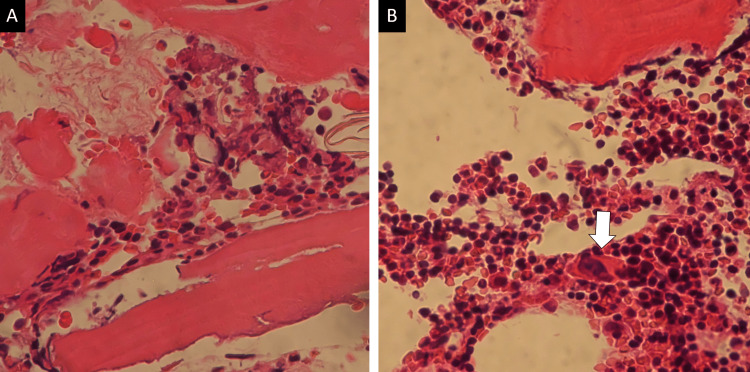
Bone marrow biopsy images before (A) and after (B) treatment. After treatment, the megakaryocyte appearance was observed (arrow) (×400, hematoxylin-eosin staining).

**Table 2:** Autoantibodies and other laboratory examinations. RF: rheumatoid factor, CCP: cyclic citrullinated peptide, ds-DNA: double-stranded DNA, SS-A: Sjogren's syndrome type A, SS-B: Sjogren's syndrome type B, MPO-ANCA: myeloperoxidase-anti-neutrophil cytoplasmic antibody, PR3-ANCA: proteinase-3-anti-neutrophil cytoplasmic antibody, HIT: heparin-induced thrombocytopenia

	On admission		Reference
Total bilirubin	0.6	mg/dL	0.4-1.5
Lactate dehydrogenase	194	IU/L	124-222
Reticulocyte	4.2	%	0.8-2.2
Haptoglobin	9	mg/dL	19-170
Thrombopoietin	0.92	fmol/mL	<0.68
Erythropoietin	32.6	mIU/mL	4.2-23.7
Vitamin B12	1,043	pg/mL	233-914
Folic acid	14.1	pg/mL	3.6-12.9
Platelet-associated Immnoglobulin G	59.7	ng/10⁷cells	0-27.6
Direct Coombs	(-)		(-)
Indirect Coombs	(-)		(-)
ANA	(-)		(-)
RF	232.9	IU/mL	<15
Anti CCP antibody	(-)		(-)
Anti ds-DNA antibody	(-)		(-)
Anti SS-A antibody	(-)		(-)
Anti SS-B antibody	(-)		(-)
MPO-ANCA	(-)		(-)
PR3-ANCA	(-)		(-)
HIT antibody	(-)		(-)
Lupus anticoagulant	(-)		(-)
Anti cardiolipin antibody	(-)		(-)
Parbovirus B19 Immnoglobulin M	(-)		(-)
Helicobacter pylori antigen	(-)		(-)

**Figure 3:**
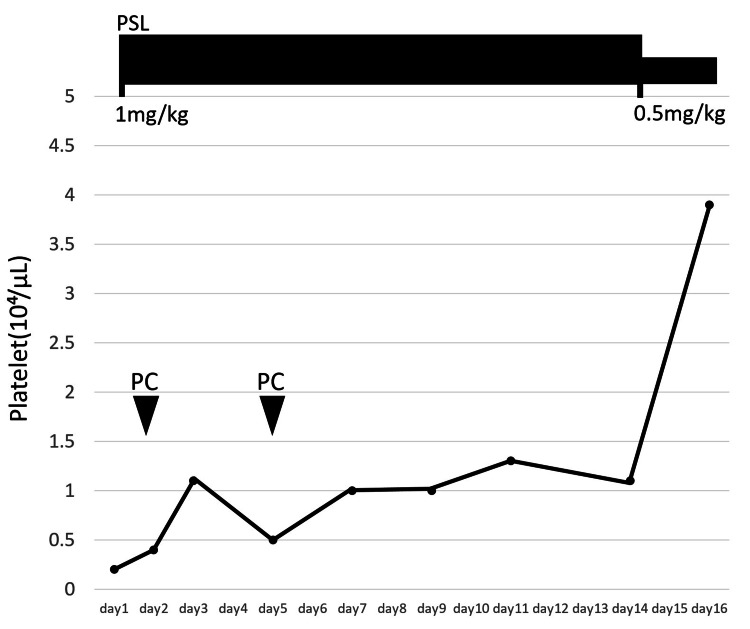
The platelet count increased to 39,000/μL on the 16th day of admission. PSL: Prednisolone, PC: Platelet Concentration

## Discussion

Several authors have reported AATP cases following autoimmune diseases (systemic lupus erythematosus, rheumatoid arthritis, and Adult-onset Still's disease) [10-13]. Only two AATP cases following AIHA were reported [7,13]. Our case is considered a rare case of AATP associated with autoimmune diseases, AIHA. 

Three findings led us to diagnose this case as Coombs-negative AIHA: Haptoglobin was markedly decreased, indicating hemolysis; There were no other coexisting hemolytic conditions such as thrombotic thrombocytopenic purpura, hemolytic uremic syndrome, or paroxysmal nocturnal hematuria; The hemolytic findings were resolved with steroids. The diagnosis would have been more specific if we had tested for red blood cell-associated immunoglobulin.

Anti-thrombopoietin (TPO) receptor antibodies were reported to inhibit the binding of TPO to the TPO receptor and suppress megakaryocyte differentiation in the bone marrow [14]. Anti-TPO receptor antibodies are frequently found in SLE patients with thrombocytopenia [15]. Anti-TPO receptor antibody-positive cases also existed in ITP patients, with higher TPO levels and poorer responses to TPO receptor agonists [16]. Previously reported TPO levels in AATP patients were considerably higher (mean ± SD = 13.7 ± 11.2 fmol/ml, n = 4), while those of ITP patients were only slightly higher (1.25 ± 0.39, n = 12) than those of healthy donors (0.55 ± 0.2, n = 20) [17]. High anti-TPO receptor antibody titers may cause a reactive increase in TPO. In our case, TPO slightly increased by 0.92 fmol/mL. Unfortunately, the measurement of anti-TPO receptor antibodies is unavailable in the usual laboratories. The limitation is that, in this case, anti-TPO receptor antibodies could not be measured. 

AATP may be caused not only by anti-TPO receptor antibodies. The defect in megakaryocyte colony-forming units (CFU-M) or cytotoxic autoantibody directed against CFU-M causes AATP [18]. Two cases of AATP showed in vitro suppression of megakaryocyte colony formation by their T lymphocytes and adherent monocytes [19]. In our case, there may have been other autoantibodies or immunological effects on megakaryocyte differentiation.

## Conclusions

AATP is a rare hematologic disorder, and AATP can be associated with autoimmune diseases, including AIHA. AATP can be misdiagnosed as other thrombocytopenic disorders such as ITP. We should consider repeat bone marrow biopsies when megakaryocytes in the bone marrow are decreased. Repeated bone marrow biopsies to monitor the number of megakaryocytes in the bone marrow would lead to better treatment, such as considering the duration of steroid administration or changing to other immunosuppressive agents.

## References

[1] Roeser A, Moulis G, Ebbo M (2022). Characteristics, management and outcome of acquired amegakaryocytic thrombocytopenia. Br J Haematol.

[2] Agarwal N, Spahr JE, Werner TL, Newton DL, Rodgers GM (2006). Acquired amegakaryocytic thrombocytopenic purpura. Am J Hematol.

[3] Nishino S, Kodaka T, Sawada Y, Goka T, Gotoh Y, Tsunemine H, Takahashi T (2018). Marked rebound thrombocytosis in response to glucocorticoids in a patient with acquired amegakaryocytic thrombocytopenia. J Clin Exp Hematop.

[4] Roy AM, Konda M, Sidarous GK, Atwal D, Schichman SA, Kunthur A (2020). Acquired amegakaryocytic thrombocytopenia misdiagnosed as immune thrombocytopenia: a case report. Perm J.

[5] El Omri H, Skouri H, Kraiem I (2000). Acquired amegakaryocytic thrombocytopenic purpura treated with intravenous immunoglobulins. Ann Med Interne (Paris).

[6] Tjon JM, Langemeijer SM, Halkes CJ (2021). Anti thymocyte globulin-based treatment for acquired bone marrow failure in adults. Cells.

[7] Hashimoto A, Fujimi A, Kanisawa Y (2013). Successful rituximab treatment for acquired amegakaryocytic thrombocytopenic purpura complicated with Coombs-negative autoimmune hemolytic anemia. Rinsho Ketsueki.

[8] Suyama T, Hagihara M, Kubota N, Osamura Y, Shinka Y, Miyao N (2021). Acquired amegakaryocytic thrombocytopenia after durvalumab administration. J Clin Exp Hematop.

[9] Simkins A, Maiti A, Short NJ, Jain N, Popat U, Patel KP, Oo TH (2019). Acquired amegakaryocytic thrombocytopenia and red cell aplasia in a patient with thymoma progressing to aplastic anemia successfully treated with allogenic stem cell transplantation. Hematol Oncol Stem Cell Ther.

[10] Iva C, Ira J.M, Robert S.K, Amy R, Jamile M.S (2010). Successful treatment of amegakaryocytic thrombocytopenia with eltrombopag in a patient with systemic lupus erythematosus (SLE). Clin Adv Hematol Oncol.

[11] Hashimoto A, Kanisawa Y, Fujimi A (2016). Thrombocytopenia and anemia with anti-c-Mpl antibodies effectively treated with cyclosporine in a patient with rheumatoid arthritis and chronic renal failure. Intern Med.

[12] Ichikawa T, Shimojima Y, Otuki T, Ueno KI, Kishida D, Sekijima Y (2019). Acquired amegakaryocytic thrombocytopenia in adult-onset Still's disease: successful combination therapy with tocilizumab and cyclosporine. Intern Med.

[13] Her MY, Kim TH, Chang HK, Lee WS, Yoo DH (2007). Successful treatment of acquired amegakaryocytic thrombocytopenia with cyclosporine in adult onset Still's disease. Rheumatol Int.

[14] Kuwana M, Okazaki Y, Kajihara M, Kaburaki J, Miyazaki H, Kawakami Y, Ikeda Y (2002). Autoantibody to c-Mpl (thrombopoietin receptor) in systemic lupus erythematosus: relationship to thrombocytopenia with megakaryocytic hypoplasia. Arthritis Rheum.

[15] Kuwana M, Kaburaki J, Okazaki Y, Miyazaki H, Ikeda Y (2006). Two types of autoantibody-mediated thrombocytopenia in patients with systemic lupus erythematosus. Rheumatology (Oxford).

[16] Jing FM, Zhang XL, Meng FL (2018). Anti-c-Mpl antibodies in immune thrombocytopenia suppress thrombopoiesis and decrease response to rhTPO. Thromb Res.

[17] Mukai HY, Kojima H, Todokoro K (1996). Serum thrombopoietin (TPO) levels in patients with amegakaryocytic thrombocytopenia are much higher than those with immune thrombocytopenic purpura. Thromb Haemost.

[18] Hoffman R, Bruno E, Elwell J (1982). Acquired amegakaryocytic thrombocytopenic purpura: a syndrome of diverse etiologies. Blood.

[19] Gewirtz AM, Sacchetti MK, Bien R, Barry WE (1986). Cell-mediated suppression of megakaryocytopoiesis in acquired amegakaryocytic thrombocytopenic purpura. Blood.

